# Understanding depression in autism: the role of subjective perception and anterior cingulate cortex volume

**DOI:** 10.1186/s13229-025-00638-4

**Published:** 2025-02-10

**Authors:** Yu Hao, Sarah Banker, Jadyn Trayvick, Sarah Barkley, Arabella W. Peters, Abigaël Thinakaran, Christopher McLaughlin, Xiaosi Gu, Daniela Schiller, Jennifer Foss-Feig

**Affiliations:** 1 Nash Family Department of Neuroscience, Icahn School of Medicine at Mount Sinai, New York, NY USA; 2 Department of Psychiatry, Icahn School of Medicine at Mount Sinai, New York, NY USA; 3 Seaver Autism Center for Research and Treatment, Icahn School of Medicine at Mount Sinai, New York, NY USA; 4 Center for Computational Psychiatry, Icahn School of Medicine at Mount Sinai, New York, NY USA; 5 Friedman Brain Institute, Icahn School of Medicine at Mount Sinai, 1470 Madison Ave 9th Fl, New York, NY 10029 USA; 6 Mindich Child Health and Development Institute, Icahn School of Medicine at Mount Sinai, New York, NY USA

**Keywords:** Autism spectrum disorder, Depression, Anterior cingulate cortex, Amygdala, Social impairments, Self-awareness, Affiliation

## Abstract

**Background:**

The prevalence of depression is elevated in individuals with autism spectrum disorder (ASD) compared to the general population, yet the reasons for this disparity remain unclear. While social deficits central to ASD may contribute to depression, it is uncertain whether social interaction behavior themselves or individuals’ introspection about their social behaviors are more impactful. Although the anterior cingulate cortex (ACC) is frequently implicated in ASD, depression, and social functioning, it is unknown if it explains differences between ASD adults with and without co-occurring depression.

**Methods:**

The present study contrasted *observed vs. subjective perception* of autism symptoms and social interaction assessed with both standardized measures and a lab task, in 65 sex-balanced (52.24% male) autistic young adults. We also quantified ACC and amygdala volume with 7-Tesla structural neuroimaging to examine correlations with self-reported depression and social functioning.

**Results:**

We found that ASD individuals with self-reported depression exhibited differences in subjective evaluations including heightened self-awareness of ASD symptoms, lower subjective satisfaction with social relations, and less perceived affiliation during the social interaction task, yet no differences in corresponding observed measures, compared to those without depression. Larger ACC volume was related to depression, greater self-awareness of ASD symptoms, and worse subjective satisfaction with social relations. In contrast, amygdala volume, despite its association with clinician-rated ASD symptoms, was not related to depression.

**Limitations:**

Due to the cross-sectional nature of our study, we cannot determine the directionality of the observed relationships. Additionally, we included only individuals with an IQ over 60 to ensure participants could complete the social task. We also utilized self-reported depression indices instead of clinically diagnosed depression, which may limit the comprehensiveness of the findings.

**Conclusions:**

Our approach highlights the unique role of subjective perception of autism symptoms and social interactions, beyond the observable manifestation of social impairment in ASD, in contributing to self-reported depression, with the ACC playing a crucial role. These findings imply possible heterogeneity of ASD concerning co-occurring depression. Using neuroimaging, we were able to demarcate depressive phenotypes co-occurring alongside autistic phenotypes.

**Supplementary Information:**

The online version contains supplementary material available at 10.1186/s13229-025-00638-4.

## Background

Depression is one of the most commonly observed co-occurring psychiatric conditions in adults with autism spectrum disorder (ASD) [1], yet the reasons behind this co-occurrence remain unclear. The lifetime prevalence of depression in ASD is estimated to be 10-49% depending on the measurement, which is significantly higher than rates in the general population [2–4]. The presence of comorbid depression may substantially impact quality of life for adults with ASD, affecting their education, employment, and satisfaction with social relations [5–7]. Understanding the phenotypes that differentiate depression and ASD could point toward new intervention paths to improve psychosocial outcomes, adaptive functioning, and overall quality of life for adults with ASD.

Depression is in general linked to social factors such as the number, quality, and reciprocity of social relationships [8]. Given that interpersonal difficulties are central to an ASD diagnosis [9], there is compelling evidence to suggest that social factors play a critical role in the etiology of depression in adults with ASD [10, 11]. However, there are mixed findings on the relationship between clinician-rated ASD symptom severity and depression [12–16]. Additionally, there may be significant variability among adults with ASD in their awareness of their social interaction successes or difficulties. Young people with ASD who are more conscious of themselves, and their social difficulties tend to experience greater emotional pain when faced with social failures, the risk of which increases when social interactions are more conflictive [17, 18]. While social deficits in ASD may contribute to depression, it remains unclear whether it is the observed social impairments or the subjective awareness of difficulties in these interactions and subsequent feelings about social connections that have a greater impact on depression in ASD individuals.

Findings on the relation between self-rated autism symptoms and depression in young adults with ASD are limited. For example, Gotham et al. (2014) found that greater self-perceived autistic impairments in young adults were associated with increased depression symptoms [19], while Murray et al. (2019) found no such relationship [20]. Other measures of self-perception of social interaction, such as perceived lack of tangible social support, feeling different from others, and low self-perceived social competence, have been associated with increased symptoms of depression with ASD [21–23]. Additionally, Day et al. (2019) found that higher self-awareness of social impairments, defined as being aware of one’s disability and differences from others, predicted depression symptoms among young adults with ASD who had average or above average IQ [24]. This evidence suggests the need for more research to differentiate between observable measures, such as clinician-rated autism symptoms, and subjective perceptions of autism symptoms and social difficulties in the development of depression.

Beyond the debate on how autism symptoms correlate with depression, less attention, despite advances in neuroimaging, has been given to how brain structures underlying autism symptoms contribute to depression in the autism population. We conducted an a priori test of brain regions previously implicated in both the ASD and depression literatures: the anterior cingulate cortex (ACC). Anatomical and functional evidence suggested that the ACC contributes to social cognition by estimating the motivation of others and dynamically updating these estimates when new evidence suggests they were incorrect (see review from Apps et al. 2016 [25]). Studies have found functional abnormalities in the ACC in individuals with ASD, either through resting-state fMRI [26] or task-based fMRI [27–30] investigating social deficits, and thinner cingulate in ASD compared with typically developed individuals [31, 32]. Less is known about how the volume of the ACC relates to ASD-related phenotypes. In addition, ACC volumetric reduction is considered a biomarker in major depressive disorder and self-rated depressive symptom severity [33–36], but its relationship to depression in ASD is not well understood. In examining whether the volumes of ACC in autistic individuals are associated with their ASD symptoms and depression, we also incorporated amygdala in our analysis. Cumulative evidence has demonstrated abnormalities in the amygdala structure and its function during social and emotional processing in autism [37–39] and a correlation between amygdala volumes and autism symptoms severity [40–45]. As the amygdala volume has frequently been studied in association with anxiety in ASD [46, 47], it can be a region of interest to contrast with the ACC to better understand the relationship between ASD phenotypes and depressive phenotypes in ASD.

Thus, the primary aim of the present study was to understand the contributions of *observed* vs. *subjective perception* of autism symptoms on the occurrence of depression in adults with ASD. We considered both clinician-rated autism symptoms and participants’ self-rated autism symptoms as a pair of contrast. Utilizing 7T structural neuroimaging, we then tested whether ACC volume was related to autism and depression symptoms while controlling for amygdala volume. The examination incorporating structural neuroimaging can help us demarcate the biological phenotypes of co-occurring depression from those of ASD and examine their relation to behavioral phenotypes.

We also conducted exploratory analyses that expanded another two paired contrasts of *observed* vs. *subjective perception* of social interactions. We first incorporated surveys to assess frequency of social contacts as an objective index of social relations, and we also assessed subjective satisfaction with social relations. It has been shown that network size in social contacts was related to depression [48], but subjective satisfaction with social relations might matters more for quality of life in the general population [49]. If self-awareness and introspection are important contributors to depression in ASD, we would expect that worse subjective satisfaction with social relations would have a greater impact than objective index of social relations on depression in the ASD population. The second pair of *observed* vs. *subjective perception* of social interaction was assessed with affiliation behaviors during a dynamic social navigation task contrasted with subjective impression of affiliation with task characters reported directly following task completion. Past research from our lab using this task found that individuals with ASD showed lower social affiliation behaviors (e.g., sharing personal information or physical contacts) than typically developing individuals [50]. Moreover, it was found that young adults in the general population with depression showed lower perceived affiliation [51]. Lower social connectedness can exacerbate life dissatisfaction [52]. In the present study, we tested how observed affiliation and subjective perception of affiliation would be related to depression in ASD. Taken together, these three paired examinations of observed vs. subjective perception indices of autism symptoms and social interaction can help us understand the role of ASD-related social impairment in the etiology of depression co-occurring in autism, as well as why autistic individuals are more prone to depression than others. Last, we explored the ACC volume role in relation to subjective indices of social interaction.

## Methods

### Participants

Participants were recruited through our local research registry, physical flyers around New York City, email listserv announcements, and word of mouth. The eligibility criteria were: (1) ages 18 to 50, (2) meeting DSM-5 criteria for ASD, (3) having an IQ over 60 (assessed by the Wechsler Abbreviated Scale of Intelligence and Wechsler Intelligence Scale for Adults), (4) having no history of neurological concerns like epilepsy or traumatic brain injury, and (5) no substance or alcohol abuse disorders nor recreational drug use. Both individuals with a prior autism diagnosis and those suspected of having autism participated in this study. We conducted a thorough diagnostic evaluation to confirm the diagnosis for all participants; ASD screening was conducted by licensed clinicians using the ADOS-2, supplemented with developmental and clinical history as needed, to inform DSM-5 criteria. We intentionally over-recruited females to ensure better representation of this underrepresented group in autism research.

Since there is no prior research on the relationship between brain structure and depression in ASD, and no studies examining the contrast between objective and subjective evaluations of social functioning, we couldn’t perform a power analysis to estimate the sample size. In total, 104 autistic individuals participated in the study and 92 participants had complete demographic and IQ measures. Among them, 40 individuals were taking SSRI/SNRI medication, which can be prescribed for depression. Among these 92 participants, 73 completed the social task and 72 underwent MRI scan. Seven participants’ MRI data was removed due to bad quality, and the final analysis included 65 sex-balanced (52.24% male) young adults with ASD (mean age = 27.32, SD = 8.77) who had average or above average IQ for volumetric analysis. Among 65 individuals who had MRI data, 26 had prior autism diagnoses. The Icahn School of Medicine at Mount Sinai’s Institutional Review Board approved the study protocol (#16-01089). All participants gave written informed consent and received compensation for their participation.

### Assessment of autism and depression

Clinical assessments of autism symptom severity and intelligence were collected in addition to self-rated autism symptom severity, self-rated depression symptoms and self-reported depression diagnoses. Demographic information and statistics of clinical assessment is shown in Table 1.

**Table 1:** Descriptive statistics of demographic and clinical symptoms in study participants (*N* = 92). Welch two-sample t-tests were performed to evaluate group differences based on self-reported depression diagnoses

	Self-reported depression Dx		
	YES (37, 40%)	NO (55, 60%)	T-test
Age	28.16 (7.88) 18.17–45.78	26.80 (9.89) 18.10–56.83	t = 1.08, *p* = 0.281
Sex	15 males 22 females	34 males 21 females	t = 2.20, *p* = 0.030
IQ	106.60 (18.78) 67–150	104.60 (17.79) 63–140	t = 0.82, *p* = 0.417
Income	4.81 (3.00) 1–9	5.36 (2.93) 1–9	t = -0.88, *p* = 0.384
Education	6.89 (1.33) 3–9	6.95 (1.27) 5–9	t = -0.19, *p* = 0.847
ADOS total	14.54 (3.32) 9–24	15.18 (4.86) 7–26	t = -0.99, *p* = 0.325
ADOS Social	8.27 (1.71) 4–13	8.35 (2.64) 4–16	t = -0.43, *p* = 0.669
BAPQ	4.27 (0.70) 2.67–5.25	3.57 (0.61) 1.56–4.69	t = 5.03, *p* < 0.0001
Zung self-rated depression scale	51.32 (9.72) 35–70	43.56 (7.02) 31–60	t = 4.60, *p* < 0.0001

Note Income levels: 1. $0 - $15,000, 2. $15,000 - $24,999, 3. $25,000 - $34,999, 4. $35,000 - $49,999, 5. $50,000 - $74,999, 6. $75,000 - $99,999, 7. $100,000 - $149,999, 8. $150,000 - $199,999. Education levels: 1. Preschool, 2. Kindergarten, 3. Elementary school, 4. Junior high school, 5. Partial high school, 6. High school graduate, 7. Partial college training/Associate’s degree, 8. Standard college/university graduate, 9. Completed graduate /professional training

#### ADOS-2 module 4

The Autism Diagnostic Observation Schedule, 2nd edition (ADOS-2 [53]), is a semi-structured diagnostic tool for autism that includes different modules for varying levels of verbal and developmental abilities. Module 4 was used in this study, which was comprised fully of verbally fluent adults. Everyone who participated in this study was assessed using the same module, with a total score range of 0–22. Therefore, we did not need to calibrate the scores. We used the total score instead of the clinical severity score, which ranges from 0 to 10, to ensure a wider range of available scores. In the following sections, we will refer to this as *clinician-rated autism symptoms*. We also obtained ADOS reciprocal social interaction subscale scores, which will be referred as *clinician-rated ASD social impairments*.

#### Broad autism phenotype questionnaire

The Broad Autism Phenotype Questionnaire (BAPQ) is a self-report questionnaire for adults that contains 36 statements. It yields subscales measuring aloofness, rigidity, and pragmatic language. This questionnaire has succeeded in meeting both sensitivity and specificity requirements for detecting the broader autism phenotype [54]. Its robust psychometric properties [54, 55]and absence of ceiling effects [56] in individuals with ASD indicate that it performs effectively in both clinical populations [57] and the general population. In the following sections, we will refer to this as *self-rated autism symptoms*.

#### Intelligence quotient

The Wechsler Abbreviated Scale of Intelligence, Second Edition (WASI-II), is a measure of cognitive ability for individuals aged 6–90 years. The Full Scale IQ (FSIQ) was calculated based on the administration of all four subtests (FSIQ-4 [58]). In addition, the Wechsler Intelligence Scale for Adults – Fourth edition (WAIS-IV [59]) is used to assess intellectual profile for people between 16 and 90 years old. It is composed of four scores and a general intelligence index. The four indexes are VCI, PRI, WMI and PSI. In our sample, 16 participants completed the WAIS and 81 participants completed the WASI. In the following sections, we will refer to these scales as *IQ*.

#### Self-reported major depressive disorder diagnosis

Depression was measured by a screening question: “Have you ever been diagnosed with any of the following psychiatric disorders?” Major Depressive Disorder (MDD) is one of the options. The response to this question is either “yes” or “no.” If participants selected “yes” for MDD, they were considered part of the depression group for this study, and vice versa. In our sample, 40% of the participants reported a diagnosis of MDD. In the following sections, we will refer to this as *self-reported depression diagnosis.*

#### Zung self-rated depression scale

The Zung self-rated depression scale consists of 20 items rated on a Likert scale of 1–4 (a little of the time, some of the time, good part of the time, most of the time) that measure the four common characteristics of current depression symptoms: the pervasive effect, the physiological equivalents, other disturbances, and psychomotor activities [60]. The scores range from 25 to 100, with 50 as clinical depression cutoff. In the following sections, we will refer to this as *self-rated depression symptoms.*

### Social interaction survey and task

#### Social relations survey

The Social Relations Survey was adapted from Quality of Life Interview developed by Lehman (1983) [49], which has two subscales. One subscale measures *objective social contacts* with items such as how often you do things with a close friend, visit someone who does not live with you, or call or text someone who does not live with you. The other subscale measures *subjective satisfaction with social relations*, including questions like: How do you feel about the things you do with other people? Are you satisfied with the amount of time you spend with other people? How do you feel about the people you see socially, the number of friendships in your life, and how you get al.ong with other people in general?

#### Social navigation task

**Fig. 1:**
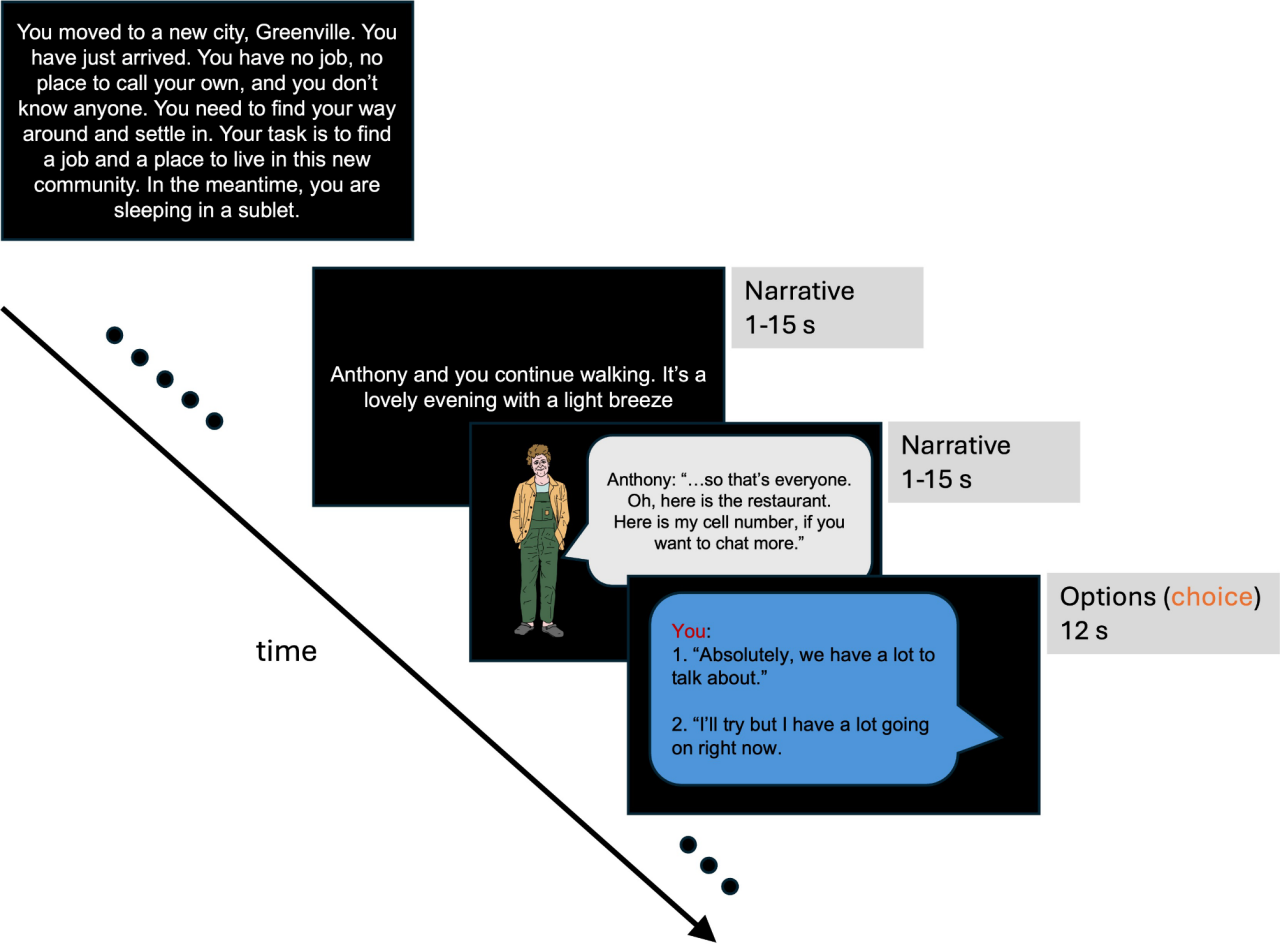
Schematic diagram of social navigation task. Participants engaged in an interactive game simulating a scenario of navigating a new town to secure employment and housing by interacting with local residents. Cartoon characters with distinct social roles (e.g., an old acquaintance or potential employer) appeared on slides, and participants selected responses via button presses, choosing between higher affiliation behavior (+ 1) or lower affiliation behavior (-1). For example, as shown in the diagram, response 1 to Anthony is counted as + 1 and response 2 to Anthony is counted as -1. In total, 30 affiliation trials were presented

In this study, participants engaged with a role-playing game designed to map out their individual social landscapes, tracing their interactive pathways with various characters [61]. The game simulated a scenario in which participants find themselves in a new town, with the objective of securing employment and housing by interacting with local residents. During gameplay, participants encountered local residents as cartoon characters on slides, with conversation occurring via text bubbles. These characters lacked a visual spatial context but possessed distinct attributes suggestive of their social roles—such as an old acquaintance or a potential employer. Participants navigated the social scenarios by choosing from two dialogue options, using a button press to dictate their responses. Some of these responses represent social affiliation behaviors with one option representing higher affiliation behavior ( = + 1) and the other option representing lower affiliation behavior (= -1). An example is shown in Fig. 1. The task also incorporated trials representing power behaviors but they were not included in our analysis. We have 5 characters in the game, and for each character, there are 6 trials of affiliation choices. In the analysis, we calculated the mean of affiliation choices across all 30 trials to represent *observed affiliation*. After completing the task, participants were asked to rate friendship or intimacy they felt with each character. We then calculated the mean of the affiliation perceptions across the 5 characters to represent *subjective affiliation*.

### Imaging acquisition and processing

Structural MRI data was acquired for all participants on a 7 Tesla whole body scanner (Magnetom, Siemens Healthcare, Erlangen, Germany). A SC72CD gradient coil was used with a single coil transmit and a 32-channel head coil (Nova Medical, Wilmington, MA, USA). A T1-weighted MP2RAGE sequence was performed on each participant, with a 0.7 mm × 0.7 mm × 0.7 mm voxel resolution. Field of view (FOV) was 225 × 183, orientation of scan was coronal, repetition time (TR) was 6000 ms and echo time (TE) was 3.62 ms. A coronal-oblique T2-weighted turbo spin echo (T2-TSE) sequence was also obtained for all participants, with a 0.43 mm × 0.43 mm × 2.0 mm voxel resolution. FOV was 222 × 177, orientation of scan was coronal, TR was 9000 ms and TE was 69 ms. Results included in this manuscript come from preprocessing performed using *fMRIPrep* 22.0.0 (RRID: SCR_016216) [62, 63], which is based on Nipype 1.8.3 (RRID: SCR_002502) [64, 65]. One T1-weighted (T1w) image was found within the input BIDS dataset. The T1-weighted (T1w) image was corrected for intensity non-uniformity (INU) with N4BiasFieldCorrection [66], distributed with ANTs 2.3.3 (RRID: SCR_004757) [67], and used as T1w-reference throughout the workflow. The T1w-reference was then skull-stripped with a *Nipype* implementation of the antsBrainExtraction.sh workflow (from ANTs), using OASIS30ANTs as target template. Brain tissue segmentation of cerebrospinal fluid (CSF), white-matter (WM) and gray-matter (GM) was performed on the brain-extracted T1w using fast (FSL 6.0.5.1:57b01774, RRID: SCR_002823) [68]. Brain surfaces were reconstructed using recon-all (FreeSurfer 7.2.0, RRID: SCR_001847) [69], and the brain mask estimated previously was refined with a custom variation of the method to reconcile ANTs-derived and FreeSurfer-derived segmentations of the cortical gray-matter of Mindboggle (RRID: SCR_002438) [70]. Volume-based spatial normalization to two standard spaces (MNI152NLin2009cAsym, MNI152NLin6Asym) was performed through nonlinear registration with antsRegistration (ANTs 2.3.3), using brain-extracted versions of both T1w reference and the T1w template. The following templates were selected for spatial normalization: *ICBM 152 Nonlinear Asymmetrical template version 2009c* [71][RRID: SCR_008796; TemplateFlow ID: MNI152NLin2009cAsym], *FSL’s MNI ICBM 152 non-linear 6th Generation Asymmetric Average Brain Stereotaxic Registration Model* [72][RRID: SCR_002823; TemplateFlow ID: MNI152NLin6Asym]. FreeSurfer (http://surfer.nmr.mgh.harvard.edu) automated segmentation of the volumes was used to extract ACC and amygdala volume. We averaged the volumes of the bilateral rostral anterior cingulate and caudal anterior cingulate to obtain the ACC volume and we averaged the bilateral amygdala volumes as amygdala volume for the subsequent statistical analysis. Information about the testing of rostral and caudal ACC separately is available in the Supplementary Materials.

### Statistical analyses

The analyses focused on assessing whether subjective perception of autism symptoms and social impairment are related to depression in the ASD population and whether ACC volume explains this relationship. We verified that all data points fell within three standard deviations of the mean, confirming that no outliers were present. We ran statistical models to address the following 4 questions (including primary and exploratory). In the introduction, we introduced 3 pairs of variables to represent *observed vs. subjective perception* of autism symptoms and social interaction. We conducted 3 multiple regression models each using a pair of the contrasting variables. To address the first question of whether clinician-rated or self-rated autism symptoms correlate with depression, we utilized a multiple regression model. This model predicted depression using both clinician-rated autism symptoms (as indicated by ADOS scores) and self-rated autism symptoms (as indicated by BAPQ scores), while controlling for age, sex, IQ, and socioeconomic status (composite score of income and education).

The second question explored whether self-reported *objective vs. subjective perception* measures of social interaction are related to depression in individuals with ASD. We applied a multiple regression model to predict depression using measures of both objective social contacts and subjective satisfaction subscales of the social relations survey, controlling for the same covariates mentioned in the first question. Then, we examined whether observed affiliation behaviors during the social interaction task or subjective perception of affiliation during the task are related to depression in individuals with ASD, again applying the same regression model with both observed affiliation and subjective affiliation included.

For our third inquiry into the role of the ACC in individuals with ASD and depression, we included ACC volume and controlled amygdala volume in our regression models to predict clinician-rated ASD symptoms (i.e., ADOS total scores and ADOS reciprocal social interaction subscale scores) and depression, respectively, controlling for the previously mentioned covariates plus intracranial volume.

Lastly, we sought to determine whether ACC volume correlates with subjective ASD symptom, social interaction and affiliation during tasks, using regression models to predict each social interaction outcome with ACC volume and amygdala volume controlled in the analyses.

In the above analyses, we used self-reported depression diagnosis first and then repeated the procedure on Zung self-rated depression symptoms. We reported standardized beta coefficients ($\:\beta\:)$ and 95% confidence interval (*CI*) to indicate effect sizes in all the analyses along with test statistics and p values. All *p* values and *CI* were corrected for multiple comparisons within a given question using the false discovery rate. We verified that our data and analyses did not have severe multicollinearity issues. The correlation among independent variables (see Supplementary Materials) and the variance inflation factor (all below 1.8) confirmed this.

## Results

### ASD with depression is related to higher subjectively perceived, but not observed, autism symptoms

**Fig. 2:**
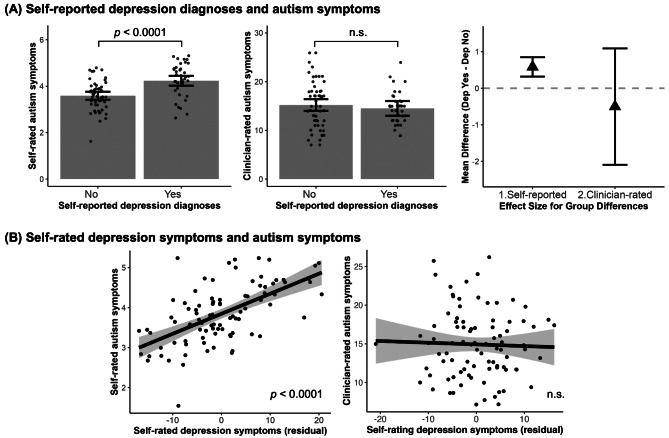
Self-reported depression relation to observed vs. subjective perception of autism symptoms. (**A**) Compared to individuals with ASD without depression, those with ASD and self-reported depression diagnoses show higher self-rated autism symptoms (subjective perception) but no difference in clinician-rated autism symptoms (observed). The triangles represent the difference between those with self-reported depression and those without self-reported depression. (**B**) scatter plots show autism symptoms relation to Zung self-rated depression symptoms with consistent findings to self-reported depression diagnoses. The graphs were made with values regressed out covariates (sex, age, IQ and SES) and show self-rated depression correlates with self-rated, but not clinician-rated, ASD symptoms. All error bars and shaded areas represent 95% confidence intervals

When both self-rated autism symptoms and clinician-rated autism symptoms were included together in the model to predict self-reported depression diagnosis, higher self-rated autism symptoms were associated with a higher chance of depression diagnosis, ($\:{\chi\:}^{2}$ = 20.36, $\:\beta\:$ = 1.28, *95% CI* = [0.52, 2.03], *p* < 0.0001), while clinician-rated ASD symptoms were not ($\:{\chi\:}^{2}$ = 0.32, $\:\beta\:$ = -0.15, *95% CI* = [-0.76, 0.46], *p* = 0.892), shown in Fig. 2A. Results for self-rated depression symptoms paralleled those for self-reported depression diagnosis, with higher self-rated ASD symptoms associated with higher depression symptoms ($\:F$ = 54.82, $\:\beta\:$ = 0.64, *95% CI* = [0.44, 0.84], *p* < 0.0001), whereas clinician-rated ASD symptoms were not ($\:F$ = 0.02, $\:\beta\:$ = -0.01, *95% CI* = [-0.21, 0.18], *p* = 0.892), shown in Fig. 2B. Controlling for prior ASD diagnosis status and/or depression medication use did not change our conclusions. In our sample, self-rated autism symptoms and clinician-rated autism symptoms were not correlated (*r* = -0.08, *p* = 0.448).

### ASD with depression is related to subjective perception but not observed indices of social interaction

**Fig. 3:**
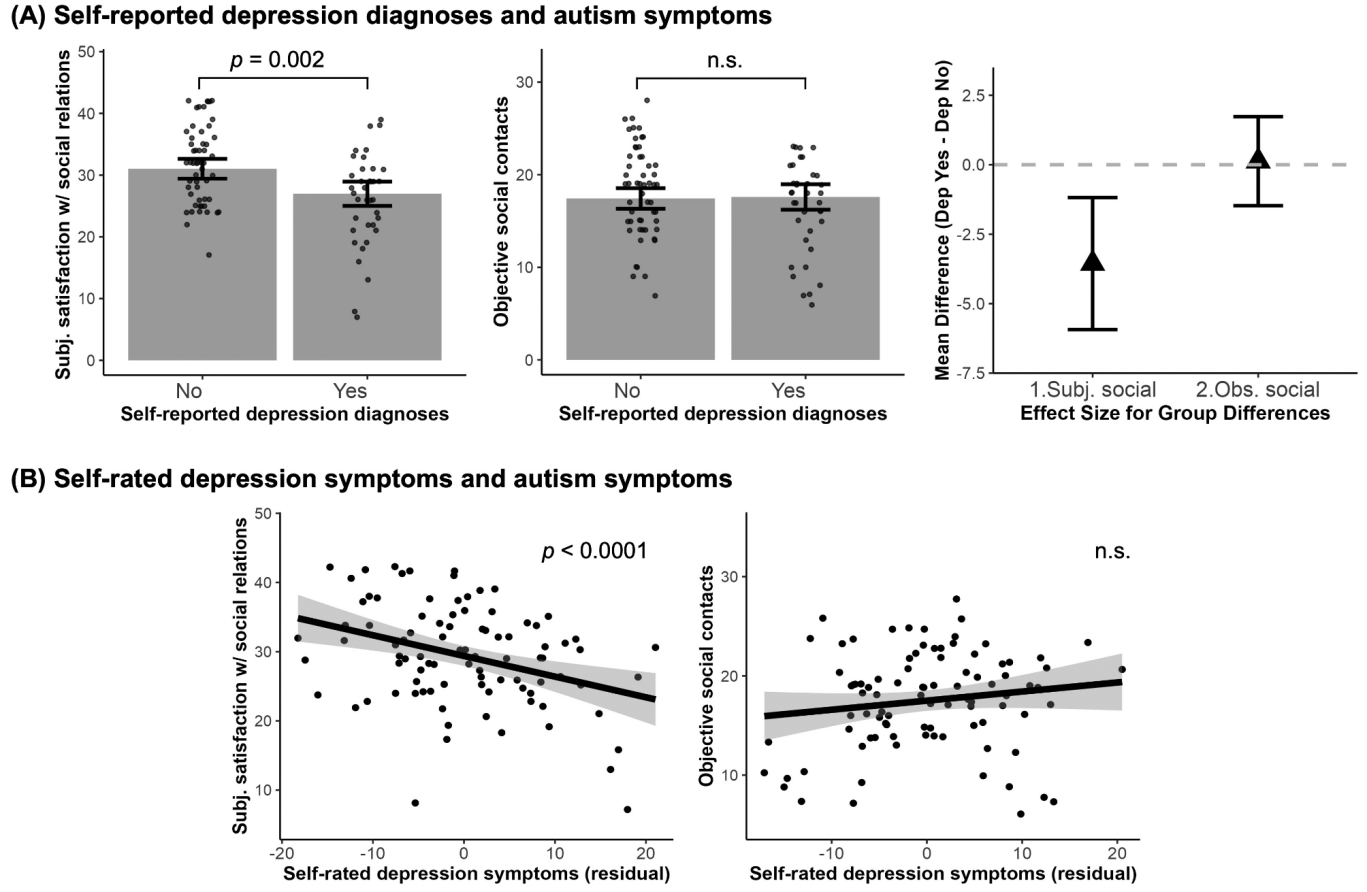
Self-reported depression relation to *observed vs. subjective perception* of social relations. (**A**) Compared to individuals with ASD without depression, those with ASD and self-reported depression diagnoses show lower subjective satisfaction with social relations but no difference in objective social contacts. The triangles represent the difference between those with self-reported depression and those without self-reported depression. (**B**) scatter plots show social relation to Zung self-rated depression symptoms with consistent findings to self-reported depression diagnoses. The graphs were made with values regressed out covariates (sex, age, IQ and SES). All error bars and shaded areas represent 95% confidence intervals

If self-reported depression was related to subjective but not observed autism symptoms, we would expect other depression-related measures to similarly show stronger effects in subjective than observed measures. To test this, we conducted exploratory analyses on another two sets of observed vs. subjective social interaction measures. When both subjective satisfaction with social relations and objective social contacts were included in the model to predict self-reported depression diagnosis, only lower subjective satisfaction with social relations was related to a higher chance of depression diagnosis ($\:{\chi\:}^{2}$ = 9.78, $\:\beta\:$ = -1.00, *95% CI* = [-1.80, -0.19], *p* = 0.002); objective social contacts were not ($\:{\chi\:}^{2}$ = 0.03, $\:\beta\:$ = 0.05, *95% CI* = [-0.68, 0.78], *p* = 0.867), as shown in Fig. 3A. Likewise, lower subjective satisfaction with social relations was related to higher depression symptoms measured by the self-rated depression scale ($\:F$ = 20.96, $\:\beta\:$ = -0.54, *95% CI* = [-0.81, -0.27], *p* < 0.0001), whereas objective social contacts were not significantly associated with depression ($\:F$ = 4.34, $\:\beta\:$ = 0.26, *95% CI* = [-0.02, 0.54], *p* = 0.080), shown in Fig. 3B.

**Fig. 4:**
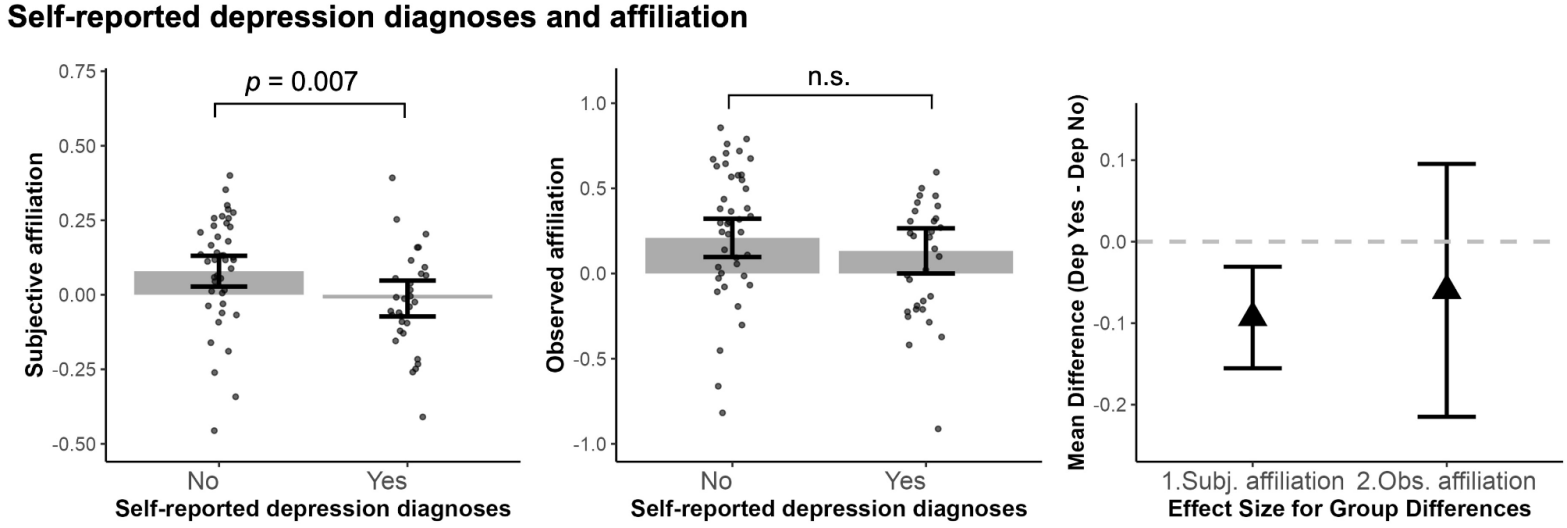
Self-reported depression relation to *observed vs. subjective perception* of affiliation in the social navigation task. Compared to individuals with ASD without depression, those with ASD and self-reported depression diagnoses show lower subjective affiliation but no difference in observed affiliation in the task. The triangles represent the difference between those with self-reported depression and those without self-reported depression. The graphs were made with values regressed out covariates (sex, age, IQ and SES). All error bars represent 95% confidence intervals

Finally, when both subjective affiliation and observed affiliation in the task were included together in the model to predict self-reported depression diagnosis, only lower subjective affiliation was related to higher probability of depression, ($\:{\chi\:}^{2}$ = 8.45, $\:\beta\:$ = -1.03, *95% CI* = [-1.92, -0.14], *p* = 0.007), while observed affiliation was not ($\:{\chi\:}^{2}$ = 0.55, $\:\beta\:$ = -0.21, *95% CI* = [-0.87, 0.44], *p* = 0.460), shown in Fig. 4. Self-rated depression symptoms were not related to both affiliation measures (all *p* > 0.13).

We further replicated these findings in an online sample of 575 young adults aged 18 to 30 (mean = 25.38, SD = 3.31) who self-reported diagnoses of autism. Of these, 375 participants (65%) self-reported a lifetime diagnosis of depression. Their depression diagnoses were found to be associated with subjective satisfaction with social relations and subjective affiliation perception during the task but not associated with corresponding observed measures. For details of methods and statistics for this replication sampling, see Supplementary Materials.

### ASD with depression is associated with larger ACC volume but not associated with amygdala volume

**Fig. 5:**
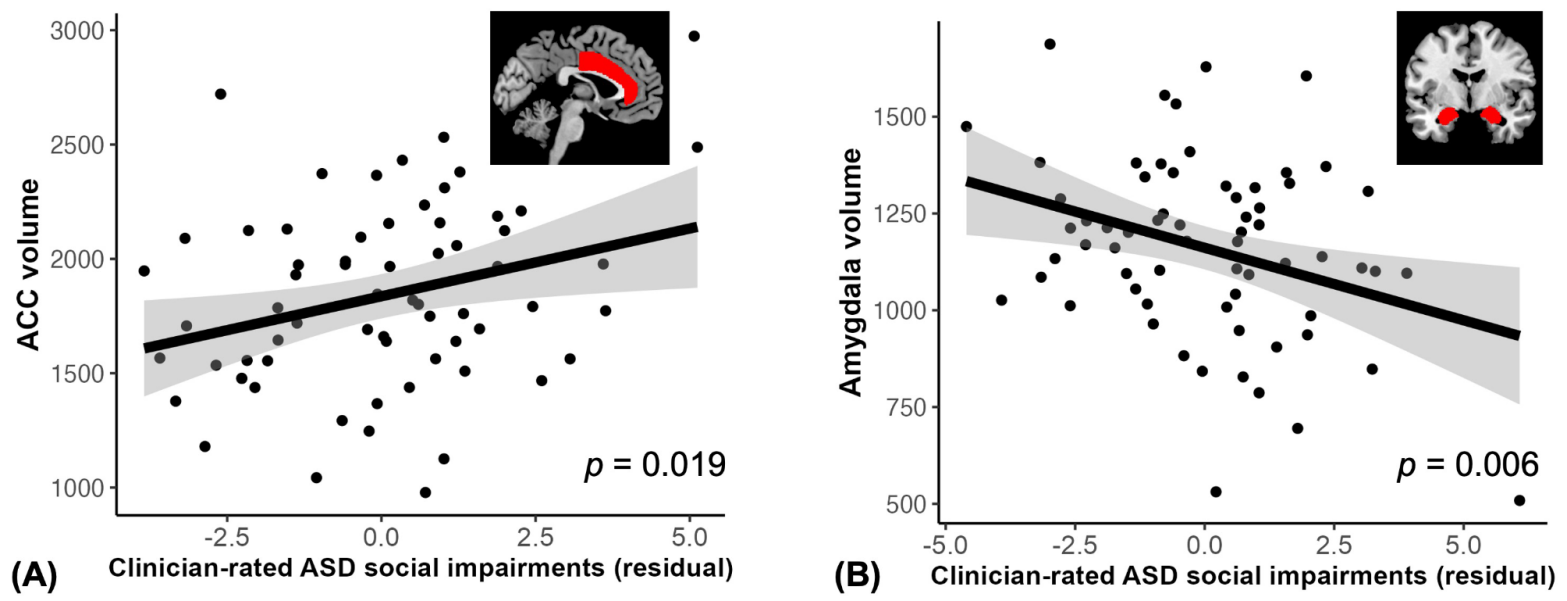
ACC and amygdala volumes relation to clinician-rated autism social impairments. Larger ACC volumes and smaller amygdala and are related to severe social interaction impairment as rated by clinicians (i.e., ADOS reciprocal social interaction subscale). The graphs were made with values regressed out covariates (sex, age, IQ, SES and intracranial volume). Shaded areas represent 95% confidence intervals

Another primary question was to examine the role of ACC volume in observed autism symptoms (i.e., clinician-rated symptoms) and depression in autism. First, we found that only smaller amygdala volume was correlated with severity of overall ASD symptoms rated by clinicians (i.e., ADOS total score, for ACC: *F* = 0.18, $\:\beta\:$ = 0.35, *95% CI* = [-0.11, 0.48], *p* = 0.148; for amygdala: *F* = 9.59, $\:\beta\:$ = -0.39, *95% CI* = [-0.67, -0.10], *p* = 0.006). Although ACC was not related to the overall clinician-rated autism symptom score, larger ACC volume was correlated with more severe clinician-rated social impairments specifically (i.e., reciprocal social interaction subscale of the ADOS, for ACC: *F* = 7.12, $\:\beta\:$ = 0.35, *95% CI* = [0.05, 0.66], *p* = 0.019, Fig. 5A; for amygdala: *F* = 8.29, $\:\beta\:$ = -0.37, *95% CI* = [-0.67, -0.07], *p* = 0.006, Fig. 5B). Controlling for prior versus new ASD diagnosis status and/or depression medication use did not change our conclusions. These findings imply that ACC volume is relevant to observable features of social impairments associated with ASD.

**Fig. 6:**
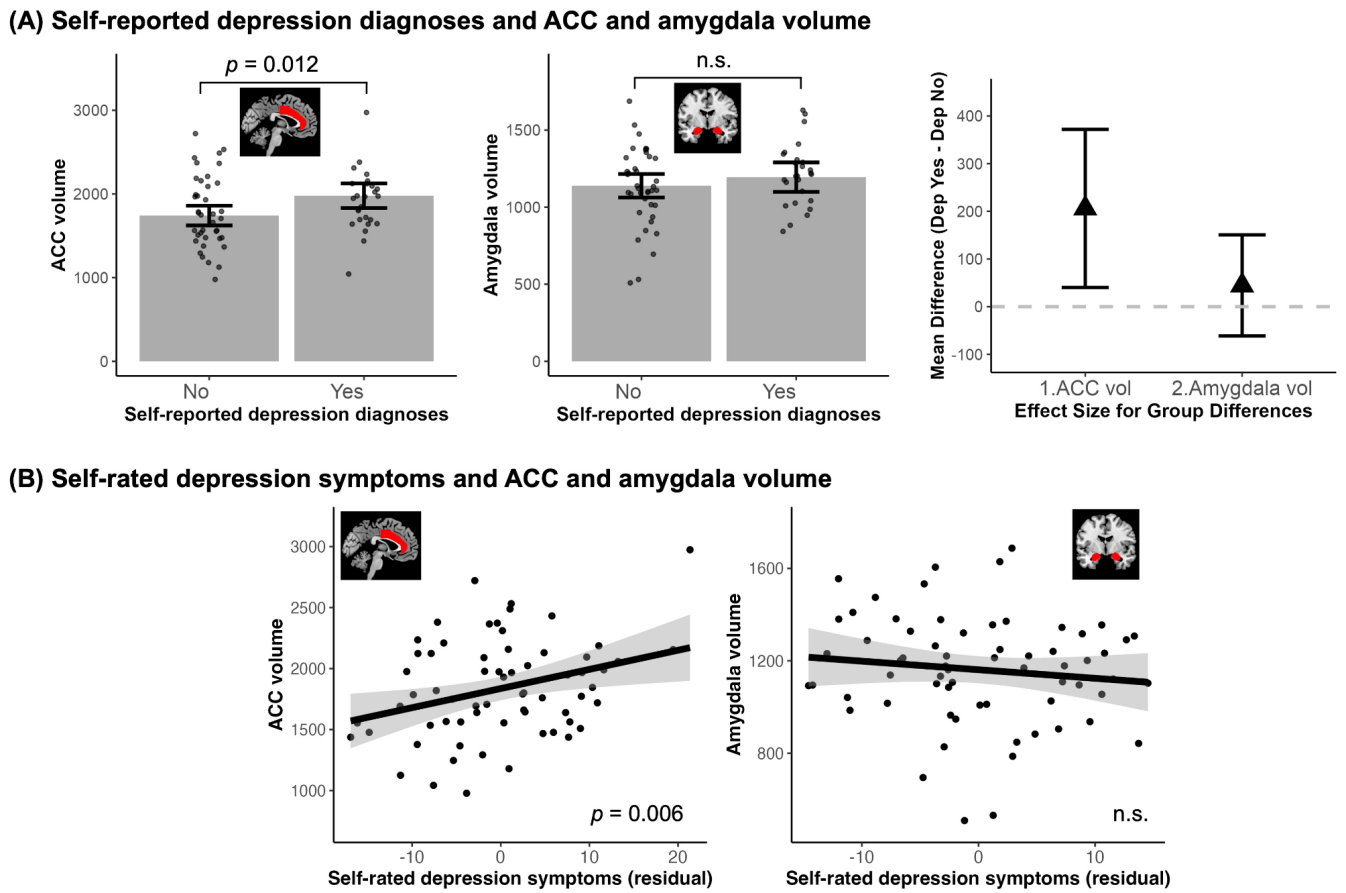
ACC and amygdala and volumes relation to self-reported depression. (**A**) Compared to individuals with ASD without depression, those with ASD and self-reported depression show larger ACC volume but no difference in amygdala volume. The triangles represent the difference between those with self-reported depression and those without self-reported depression. (**B**) scatter plots ACC and amygdala volumes relation to Zung self-rated depression symptoms with consistent findings to self-reported depression diagnoses. The graphs were made with values regressed out covariates (sex, age, IQ, SES and intracranial volume). All error bars and shaded areas represent 95% confidence intervals

Next, we analyzed if ACC volume was related to depression in our ASD sample. We found that larger ACC volume was associated with higher chance of self-reported depression diagnosis ($\:{\chi\:}^{2}$= 6.33, $\:\beta\:$ = 0.89, *95% CI* = [0.00, 1.78], *p* = 0.012); amygdala volume, however, was not predictive of self-reported depression diagnoses ($\:{\chi\:}^{2}$= 0.76, $\:\beta\:$ = 0.28, *95% CI* = [-0.47, 1.03], *p* = 0.384), shown in Fig. 6A. Since there was an extreme data point for ACC volume in the ASD with depression group, we also ran 1000 bootstrap iterations on the relation between ACC volume and self-reported depression diagnoses, and found consistent results ($\:\beta\:$ = 0.89, 95% CI = [0.24, 3.04]). Removing the highest ACC volume in the depression group also yielded consistent findings. Likewise, larger ACC volume was also associated with higher self-rated depression symptoms (*F* = 9.57, $\:\beta\:$ = 0.41, *95% CI* = [0.10, 0.71], *p* = 0.006) but amygdala volume did not show a significant relationship (*F* = 0.76, $\:\beta\:$ = -0.12, *95% CI* = [-0.41, 0.17], *p* = 0.384), shown in Fig. 6B. Controlling for prior ASD diagnosis status and/or depression medication use did not change our conclusion.

### Larger ACC volume is related to greater subjective autism symptoms and worse subjective satisfaction with social relations

**Fig. 7:**
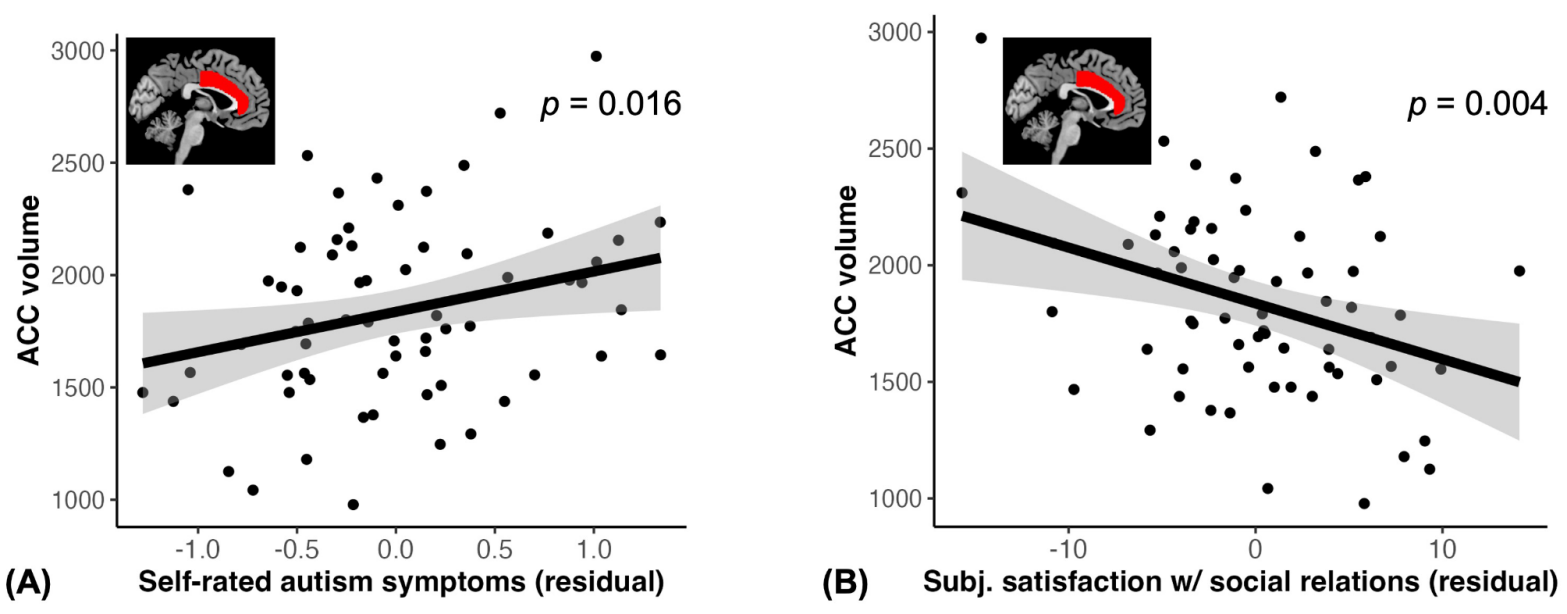
ACC volume relation to subjective autism symptom and social relations. Larger ACC volume is related to higher self-rated autism symptoms (**A**) and worse subjective satisfaction with social relations (**B**). The graphs were made with values regressed out covariates (sex, age, IQ, SES and intracranial volume). Shaded areas represent 95% confidence intervals

Extending our exploratory tests of ACC’s role, we found that ACC volume was related to self-rated autism symptoms (F = 6.17, $\:\beta\:$ = 0.31, 95% CI = [0.06, 0.57], *p* = 0.016, Fig. 7A) and subjective satisfaction with social relations (F = 10.35, $\:\beta\:$ = -0.36, *95% CI* = [-0.62, -0.10], *p* = 0.004, Fig. 7B). Amygdala volume, controlled in the analyses alongside ACC, was not a significant predictor of self-rated autism symptoms or subjective satisfaction with social relations (all *p* > 0.8). Both ACC and amygdala volumes were not related to subjective perception of affiliation. These findings suggest that the ACC may be sensitive to capturing subjective social interaction difficulties associated with depression in ASD. In contrast, the amygdala, though it was related to observed symptoms of ASD, was not associated with self-reported depression or related subjective measures of social relations and autism symptoms.

## Discussion

Although a high prevalence of depression is observed in the ASD population, the underlying mechanisms remain unknown. It is unclear whether depression is due to the observed autism symptoms related to social deficits or to individuals’ subjective introspection regarding their social impairment. What brain structures may contribute to this effect remains elusive. Investigating this question can help us understand why some individuals with ASD develop depression while others do not, potentially revealing both risk and protective factors. Our findings indicate that levels of clinician-rated ASD symptoms, objective social contacts, and observed affiliation behavior during social interaction tasks did not differ between individuals with ASD who did or did not report a history of depression nor associate with self-rated depression symptoms at the time of participation. However, compared to individuals with ASD without depression, those ASD individuals with self-reported depression diagnoses and high level of self-reported depressive symptoms exhibited heightened self-rated autism symptoms, worse satisfaction with social relations, and diminished subjective affiliation with social characters during a social navigation task. ACC volume was related to all major factors investigated here: autism symptoms, depression, and subjective perception of social difficulties.

Expanding on findings from Day et al. (2019) in young adults with ASD and low support needs, which indicated that self-awareness of social impairment is related to depression [24], our study expanded self-evaluation of social functioning to include three measures: self-rated autism symptoms, subjective satisfaction with social relations, and subjective affiliation during social navigation task. Contrary to Day et al. (2019) and a few other studies [16, 24] that found depression to be associated with less severe autism symptoms as assessed by clinicians as well as a few studies that found depression to be associated with more severe clinician-rated autism symptoms [12–14], we did not find a relationship between clinician-rated ASD symptoms and depression. Consistent with a few other studies, however, we found higher self-rated autism symptoms related to depression in our autism sample [12, 19, 73]. Our sample also showed no relation between clinician-rated and self-reported ASD symptoms. Because our sample was restricted to a group of autistic adults with average or above average IQ, our null relationships may only reflect patterns in the segment of the autism spectrum who were able to participate in our assessments. Nevertheless, our findings might imply both common and distinct mechanisms underlying externally observed autism symptoms and self-perception of autism symptoms.

Using structural neuroimaging, we were able to dissect these common and distinct mechanisms. Interestingly, larger ACC volume, which was associated with severity of autism symptoms rated by clinicians and participants’ self-awareness of autism symptoms, also correlated with a higher likelihood of self-reported lifetime depression diagnosis, greater self-rated current depression symptoms, and worse subjective satisfaction with social relationships. On the other hand, amygdala volume related only to clinician-rated autism symptom severity and was not related to self-ratings of autistic traits, depression, or subjective perception of social behaviors. The amygdala has been an important region studied in the autism literature, given its association with emotional and social processing [38, 74, 75]. While some studies found that larger amygdala volume was associated with more severe ASD symptoms [42–44], we found, consistent with a few other studies [40, 41] that reduced amygdala volume is associated with more severe clinician-observed ASD symptoms. Therefore, despite amygdala volume not relating to depression, it served as a unique control region in examining the role of the ACC on depression and social impairments in ASD.

Prior studies reported autism symptom severity in association with ACC functional abnormalities in both social-related tasks [27–29] and resting-state functional connectivity [26]. When investigating ACC structure, one study found that individuals with autism who had thinner ACC tended (*p* < 0.1) to have lower social responsiveness [31] and another study found that reduced white matter volume was related to more social awareness deficits in autism [32]. We contribute to the literature by demonstrating a significant relationship between ACC volume and social reciprocity as rated by clinicians. Moreover, we specifically showed that larger ACC was related to subjective measures including self-rated autism symptoms, self-reported lifetime depression diagnoses, self-rated depression symptom severity, and subjective satisfaction with social relations. As the ACC plays a role in estimating and updating social motivation when interacting with others [25], the ACC might serve as a hub connecting depression with self-rated autism symptoms severity and social satisfaction. It may reflect introspection and awareness of autistic symptoms, suggesting that the sources of social (dis)satisfaction and depression symptoms are interconnected.

In summary, ACC volume is associated with self-reported depression and core ASD symptoms, particularly clinician-rated reciprocal social interaction. Given amygdala volume was related to observed – but not self-reported - autism symptoms and not to depression, it may be that depression emerges in the context of increased perception of social difficulties within ASD rather than as a function of social difficulties themselves. Our exploratory analyses further demonstrated the role of subjective perception of social interaction (social relation satisfaction and subjective affiliation) in self-reported depression, which were also replicated with an online sample with different demographic distributions. It is important to note that although objective social contacts and observed affiliation behaviors are significantly lower in autism compared with typically developing individuals [50, 76, 77], they did not contribute to depression in our samples. Future studies can examine neural pattern-based ASD subtypes based on our findings to differentiate the neural substrates of observed vs. subjective manifestations of social interaction difficulties and examine their relations to depression.

Our findings are readily translatable for future studies in longitudinal samples. Future studies can examine the association between progression of depression symptoms and changes in self-rated ASD symptoms as well as satisfaction in social relationships in order to determine the directional influences of ASD symptoms, self-perception and depression. Future studies could also examine longitudinal tracking of self-reported autism symptoms, social satisfaction, etc. overtime in thinking about risk, prevention and intervention. Moreover, feelings of loneliness can be an important mediator of social communication deficits in ASD and in the development of depression (see meta-analysis [78]); future studies can examine how feeling of loneliness and introspection of social behaviors interact in this association. Finally, as our study only considered individual factors, future research should examine the interaction between individual factors and environmental stressors [79], an important risk factor for depression across populations, in the context of comorbid depression in ASD (for full reviews, see [17, 80, 81]).

### Limitation

One key limitation is the cross-sectional study design. Longitudinal studies are necessary to determine the directionality of the relationships we observe. Additionally, the study did not include individuals with an IQ below 60 as it required participants to read and to express their experiences, through button presses and written responses, interacting with social task characters and self-reporting on depression and social interaction. These requirements exclude many individuals on the spectrum, and risk factors for co-occurring depression should also be explored for these groups. In this study, we utilized self-reported depression indices instead of clinically ascertained MDD status. There is some evidence showing that presentation of depressive symptoms may be different in individual with ASD compared with typically developing individuals, such as more insomnia or restlessness [82–86]. However, there is lack of data regarding the degree to which self reports and clinician diagnoses converge regarding MDD in ASD [84]. Given rates of depression are much higher when participants with ASD report their own depressive symptoms [3], our current study focused on self-report of depression. Future study should incorporate depression measures from both self-report and clinician diagnosis and clarify both current symptoms and past episodes to provide a comprehensive view of the relation between depression and social functioning in ASD. Future study should also evaluate whether the contribution of subjective perception of social behaviors to depression in autism replicates or diverges in non-autistic samples. Finally, the findings related to brain structure are limited by sample size, highlighting the need for replication in larger samples [87].

## Conclusions

In summary, our findings suggest that greater subjective perception of social interaction difficulties and enlarged ACC may serve as potential markers indicating risk for depressive disorders in ASD. Given the link between depressive disorders, reduced adaptive function, and increased suicide risk in ASD, understanding the emergence of and risk factors associated with depression in ASD may yield significant public health benefits, including improvements in psychosocial outcomes and adaptive functioning. In the future, clinicians may be able use features identified in this study—more self-reported social impairments and poorer perceived social satisfaction—to identify individuals with ASD who are at risk for developing depression. This approach may also benefit the characterization and diagnosis of depression in ASD and facilitate identifying effective treatments.

## Electronic supplementary material

Below is the link to the electronic supplementary material.

Supplementary Material 1

## Data Availability

No datasets were generated or analysed during the current study.
