# Supplementary material for: Understanding depression in autism: the role of subjective perception and anterior cingulate cortex volume
Source: Mol Autism. 2025 Feb 10;16:9. doi: 10.1186/s13229-025-00638-4 (PMC11812218; doi:10.1186/s13229-025-00638-4)
Supplement: Supplementary file 1 — Supplementary Material 1 [file 13229_2025_638_MOESM1_ESM.pdf]

Supplementary Materials

Correlation table N = 92 – all surveys

	age	sex	IQ	SES	ADOS	ADOS social	BAPQ	Zung	Dep dx	LSR_obj	LSR_sub
age											
sex	-0.07										
IQ	-0.12	0.09									
SES	0.37***	0.03	0.39***								
ADOS	0.11	-0.21*	-0.09	-0.15							
ADOS Social	0.11	-0.05	-0.04	-0.06	0.85***						
BAPQ	0.02	0.24*	0.25*	0.11	-0.08	0.08					
Zung	0.07	0.20	-0.05	-0.09	-0.04	0.05	0.62***				
Dep dx	0.07	0.23*	0.06	-0.07	-0.07	-0.02	0.47***	0.42***			
LSR_obj	0.18	0.02	0.07	0.18	-0.05	0.06	-0.27**	-0.11	-0.17		
LSR_subj	-0.00	-0.11	0.01	0.09	0.06	-0.10	-0.52***	-0.44***	-0.39***	0.58***	

Correlation table – social navigation task (N = 73) and brain data (last two rows N = 65)

	age	sex	IQ	SES	ADOS	ADOS social	BAPQ	Zung	Dep dx	LSR_obj	LSR_sub	Affil obj	Affil subj	Amyg vol
age														
sex	-0.09													
IQ	-0.06	0.03												
SES	0.34**	0.10	0.39***											
ADOS	-0.04	-0.21	0.04	-0.23*										
ADOS Social	0.01	0.00	0.05	-0.07	0.83***									
BAPQ	0.05	0.31**	0.27*	0.22	0.03	0.17								
Zung	0.04	0.17	-0.05	-0.02	-0.03	0.10	0.62***							
Dep dx	0.08	0.15	0.06	0.04	-0.03	0.00	0.48***	0.37**						
LSR_obj	0.11	0.03	0.05	0.08	-0.13	0.01	-0.20	-0.02	-0.19					
LSR_subj	-0.08	-0.12	-0.03	-0.08	-0.04	-0.19	-0.48***	-0.40***	-0.41***	0.49***				
Affil_obj	-0.04	-0.12	0.04	0.05	-0.15	-0.11	-0.35**	-0.26*	-0.23	0.20	0.23*			
Affil_subj	-0.10	-0.12	0.16	-0.10	0.07	-0.04	-0.26*	-0.23	-0.32**	-0.19	0.19	0.36**		
Amyg vol	0.35**	-0.07	0.00	0.21	-0.21	-0.19	0.03	-0.03	0.17	0.06	0.02	-0.13	-0.08	
ACC vol	-0.07	-0.22	0.03	0.12	0.13	0.24*	0.12	0.15	0.17	-0.03	-0.24*	0.08	0.00	0.20

Note: * $p < 0.5$; ** $p < 0.01$; *** $p < 0.001$. Abbreviations: ADOS = the total score from the Autism Diagnostic Observation Schedule; ADOS social = ADOS reciprocal social interaction subscale scores; BAPQ = the total score of the Broader Autism Phenotype Questionnaire; Zung = the total score of Zung self-rated Depression Scale; Dep dx = self-reported history of depression diagnosis; LSR_obj = objective social contacts subscale of Lehman social relation survey; LSR_obj = subjective satisfaction with social relations subscale of Lehman social relation survey; Affil_obj = affiliation behavior captured by our naturalistic experiment of social navigation task; Affil_subj = subjective perception of affiliation with task characters; Amyg vol = averaged bilateral amygdala volume; ACC vol = averaged bilateral ACC volume (combined rostral and caudal ACC).

Testing of rostral and caudal ACC

We also tested rostral and caudal ACC separately and found consistent results mirroring those, presented in the main text, for ACC analyzed as a whole. Specifically, both rostral ($p = 0.006$) and caudal ($p = 0.046$) ACC volume are related to the ADOS reciprocal social interaction subscale. Rostral ACC is related to BAPQ score ($p = 0.017$) and caudal ACC follows a similar trend ($p = 0.067$). Moreover, both rostral and caudal ACC volume are related to self-reported depression diagnoses ($p = 0.013$ and $p = 0.029$, respectively) as well as the Zung self-rated depression scale (both $p < 0.01$).

Replication sample

We further replicated, in an online sample, the exploratory analyses of our social relation survey and task affiliation behavior and perception in relation to self-reported history of depression diagnosis in autism.

ASD participants were recruited through Simons Powering Autism Research (SPARK)'s Research Match, supported by Simons Foundation, through the Simons Foundation Autism Research Initiative (SFARI). All SPARK participants self-report a professional diagnosis of ASD for themselves (18 years old and independent) or their offspring (under 18 years of age, or dependent adults). The eligibility criteria were: (1) ages 18 to 30, (2) Social Communication Questionnaire > 9 (SCQ, Rutter et al., 2003). Among 956 participants who consented, 675 individuals completed all surveys and social navigation task. The sample size after excluding 100 participants with an "ASD validity flag" identified in the SPARK data release was 575 (mean age = 25.38, SD = 3.31, 409 females). Of these, 375 participants (65%) self-reported a lifetime diagnosis of depression. This data collection project was supported by a grant by Simons Foundation (Grant No. 877761, Foss-Feig, Gu, Schiller). The Icahn School of Medicine at Mount Sinai's Institutional Review Board approved the study protocol (#22-00171).

Participants completed a revised version of the *social navigation task*, where instead of securing employment, participants started a new school and needed to find their way around and settle in. Their task was to join a club and find a locker at their new school. The task structure, in terms of number and types of interactions and roles of characters were design to parallel the original task exactly. We used the same regression model as in our paper, where self-reported depression diagnoses were predicted by observed affiliation behaviors (captured in the task) and subjective perception of affiliation (post-task survey) and other covariates including sex, age, SES, and IQ. We found that self-reported depression diagnoses were associated with subjective character affiliation (χ^2 = 5.08, β = -0.21, 95% CI = [-0.03, -0.39], p = 0.024) but not associated with observed affiliation during task interactions (χ^2 = 0.03, β = 0.02, 95% CI = [-0.17, 0.21], p = 0.852). See Fig. S1(A) for the effect size visualization for group differences.

We also replicated the finding of subjective satisfaction with social relations, using the measure which was identical to the one used in imaging sample. We found that self-reported depression diagnoses were associated with subjective satisfaction with social relations (χ^2 = 17.37, β = -0.44, 95% CI = [-0.23, -0.66], p < 0.0001), but not with objective social contacts (χ^2 = 0.07, β = -0.03, 95% CI = [-0.23, 0.17], p = 0.799). See Fig. S1(B) for the effect size visualization for group differences.

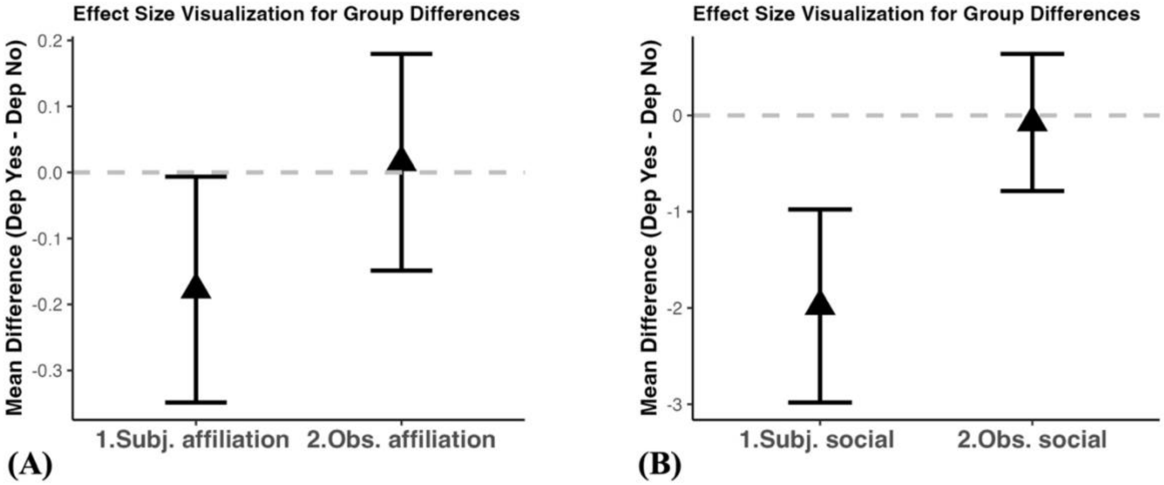

Figure S1. Effect size visualization for group differences. (A) Self-reported depression group differences in subjective affiliation (subj. affiliation) and observed affiliation (obj. affiliation). (B) Self-reported depression group differences in subjective satisfaction with social relations (subj. social) and objective social contacts (obj. social). The triangles represent the observed difference between those with self-reported depression and those without self-reported depression, with 95% confidence intervals. The graphs were made with values regressed out covariates (sex, age, IQ and SES).
